# Formation of Dual-Scale TiC Reinforcements Under Different Energy Densities and Their Influence on the Properties of Laser Metal Deposited 316L Composites

**DOI:** 10.3390/ma19184027

**Published:** 2026-09-21

**Authors:** Heng Zhu, Quan Zhu, Weijie Xia, Chao Lu, Xiaoli Cui, Yan Yin, Yutong Gao, Ruihua Zhang, Xiaoan Zeng, Wenqing Shi, Di Tie

**Affiliations:** 1 School of Materials Science and Engineering, Guangdong Ocean University, Yangjiang 529500, China; 2 School of Materials Science and Engineering, Lanzhou University of Technology, Lanzhou 730050, China; 3 Yangjiang Hardware Knife Cut Industrial Technology Research Institute, Yangjiang 529500, China; 4 School of Intelligent Manufacturing, Luoding Polytechnic, Luoding 527200, China

**Keywords:** laser melting deposition, 316L stainless steel, composite material, microstructure, corrosion resistance

## Abstract

Laser melting deposition (LMD) was employed to fabricate 5.0 wt.% TiC-reinforced 316L composite coatings, and the role of laser power in governing microstructure evolution and performance was systematically investigated. Particular emphasis was placed on the laser-power-dependent evolution mechanism of TiC, covering the coexistence of retained undissolved TiC and fine reprecipitated nanoscale TiC phases; critically, this work clarifies their combined regulating effects on hardness, wear, and corrosion behavior, advancing the understanding of structure-property correlation for LMD 316L/TiC composites. The results show that increasing laser power markedly altered melt-pool dynamics, particle dissolution, and interfacial heterogeneity, thereby regulating the process–microstructure–property relationship of the coatings. Owing to the synergistic effects of residual TiC reinforcement, fine-particle dispersion, and microstructural refinement, the P14 and P16 coatings exhibited the highest hardness, reaching ~320–330 HV. However, the best wear resistance was achieved at moderate laser power, indicating that excessive hardening did not necessarily lead to optimal tribological performance because of increased local heterogeneity and particle detachment tendency. Electrochemical results further revealed that corrosion resistance was jointly controlled by passive-film stability and particle/matrix interfacial activation. Among all samples, P12 showed the most favorable overall corrosion performance, reflecting an optimized balance between microstructural uniformity and interfacial stability. These findings demonstrate that appropriate laser power is critical for simultaneously optimizing strengthening and corrosion resistance in the LMD-fabricated 316L/TiC composite.

## 1. Introduction

316L austenitic stainless steel contains relatively high levels of Cr and Ni, together with a certain amount of Mo, which enables the formation of a relatively stable passive film in a variety of corrosive media. Owing to this characteristic, 316L stainless steel has been widely used in marine engineering, chemical equipment, nuclear power structural components, biomedical devices, and aerospace applications [1,2,3,4]. However, monolithic 316L stainless steel still suffers from insufficient service stability under complex environments involving the coupling of corrosion and mechanical loading. Under conditions such as particle erosion, impingement, friction-induced wear, and cyclic loading, surface damage and local failure can readily occur [5,6,7,8]. In the context of additive manufacturing, thermal cycling, porosity, residual stress, elemental segregation, and grain morphology may further affect the service stability of the material [5,9]. Therefore, improving the hardness and overall service reliability of 316L stainless steel while retaining its corrosion resistance remains a critical issue for its engineering applications [10].

Laser melting deposition (LMD) is an additive manufacturing technique belonging to the directed energy deposition family [11,12]. In this process, synchronized powder feeding and high-energy laser irradiation are used to produce a metallurgically bonded deposited layer on a substrate surface or along a predetermined path. LMD offers several advantages, including high material utilization, strong capability for local shaping, and suitability for repair and functionally graded manufacturing [11,12,13]. Compared with conventional casting and powder metallurgy, the rapid heating and solidification inherent to the LMD process are beneficial for the formation of refined microstructures and can, to some extent, improve the distribution of reinforcement particles. These characteristics provide a promising opportunity for fabricating high-hardness and wear-resistant coatings on 316L stainless steel surfaces while maintaining excellent corrosion resistance.

Particle-reinforced metal matrix composites offer an effective strategy for improving the performance of 316L stainless steel [14,15,16]. Among various ceramic reinforcements, TiC has attracted particular interest because of its high melting point, high hardness, high elastic modulus, good chemical stability, and acceptable wettability with steel matrices. These characteristics enable TiC to provide multiple strengthening effects, including particle strengthening, grain refinement, and load transfer [17,18,19,20]. Accordingly, TiC/316L composites have been extensively investigated in recent years. Zhai et al. [21] and AlMangour et al. [18] systematically studied the influence of TiC particles on the microstructure and properties of 316L stainless steel fabricated by selective laser melting (SLM), showing that TiC can effectively improve the strength, hardness, and wear resistance of the alloy. However, the strengthening effect is strongly dependent on processing parameters, porosity, and TiC content. In particular, Zhai et al. [21] demonstrated that laser power plays a critical role in determining the relative density of SLM-fabricated samples. At low laser powers, pores associated with incomplete melting were readily observed. They further reported that the transient melt-pool temperature during SLM can exceed the melting point of TiC, promoting the partial melting of micron-sized TiC particles and the formation of Ti–C liquid clusters. Under ultrahigh cooling rates, in situ precipitation of nanoscale TiC occurs, while the combined effects of micron-sized and in situ nanoscale TiC contribute to grain refinement, precipitation strengthening, and dislocation strengthening, thereby markedly enhancing the mechanical properties of 316L while retaining reasonable ductility [21].

Similar strengthening effects have also been reported in laser-cladded stainless steel systems. Zhu et al. [22] prepared TiC-reinforced AISI 410 martensitic stainless steel coatings with different TiC contents and found that the improved properties originated from the synergistic effects of microstructural refinement, nanoscale precipitation strengthening, and second-phase strengthening. Nevertheless, in 316L austenitic stainless steel, the enhancement in mechanical properties is still often restricted by the non-uniform distribution of reinforcing phases and insufficient interfacial bonding. To overcome these limitations, Song et al. [23] and Liu et al. [8] fabricated 316L-based composites reinforced by ex situ added TiC and in situ formed TiC using laser directed energy deposition (LDED). Their results showed that, compared with ex situ TiC reinforcement, in situ generated TiC can significantly reduce defects, improve the uniformity of reinforcement distribution, and effectively suppress anisotropy. This suggests that in situ formation of reinforcing phases is advantageous for improving the mechanical performance of metal matrix composites and mitigating the interfacial incompatibility commonly associated with externally added particles.

The importance of processing parameters has also been highlighted in related laser cladding studies. Zhang et al. [24] fabricated in situ (TiC + TiB)-reinforced 316 stainless steel composite coatings by adding Ti and B_4_C powders and demonstrated that laser power exerts a pronounced non-monotonic influence on the extent of in situ reaction, the morphology and distribution of reinforcing phases, and the final coating properties. Specifically, the coating hardness first increased and then decreased with increasing laser power, whereas the wear rate showed the opposite trend, indicating the existence of an optimal processing window. Gao et al. [17] also reported that TiC can refine grains, weaken texture, and enhance hardness in CoCrFeNi high-entropy alloy composite coatings, while excessive TiC addition may deteriorate wear resistance because of particle spallation and aggravated three-body wear. In addition to mechanical performance, TiC also affects the corrosion behavior of stainless steel-based composites. Zhang et al. [25] investigated the effect of TiC content on the corrosion resistance and mechanical properties of laser metal-deposited 316L stainless steel and found that a TiC content of 0.3 wt.% led to the best overall performance. The improved corrosion resistance was attributed to the synergistic contributions of grain refinement, an increased fraction of low-Σ coincidence site lattice grain boundaries, reduced Cr depletion, and the formation of a more stable passive film, while the enhanced mechanical properties resulted from refined grains, a more uniform distribution of reinforcing phases, and favorable interfacial interactions between TiC nanoparticles and the matrix. Overall, the above studies demonstrate that TiC-reinforced 316L composites exhibit considerable potential for engineering applications. However, their microstructural evolution and resultant properties are highly sensitive to processing conditions, especially laser-related parameters.

Existing studies have demonstrated that TiC particles can improve the strength, hardness, and wear resistance of 316L-based composites. However, the final microstructure and properties are strongly affected by the dispersion state of TiC particles, the quality of particle/matrix interfacial bonding, and the melting/decomposition behavior induced by laser heat input [20,26]. Nevertheless, three issues remain insufficiently addressed in the current literature. First, most previous studies have evaluated performance mainly from the perspectives of TiC content or macroscopic forming quality, whereas limited attention has been paid to the correlation among laser-power-induced variations in heat input, particle distribution, and corrosion behavior at a fixed TiC content. Second, improvements in hardness and corrosion resistance are not always synchronized. Higher hardness may be accompanied by particle agglomeration, interfacial defects, or elemental segregation, which can adversely affect corrosion performance. Finally, grain size, grain boundaries, TiC particles, and the formation of Cr/Mo-enriched passive films are mutually coupled, and no single electrochemical parameter is sufficient to comprehensively assess the merits of a given processing condition.

This work establishes a process–microstructure–property framework for TiC-reinforced 316L coatings by clarifying how laser-power-dependent thermal input governs the balance between retained undissolved TiC and reprecipitated nanoscale TiC, and how this heterogeneous particle architecture simultaneously affects strengthening, wear resistance, and corrosion behavior. In particular, the subtle discrepancy between EIS- and polarization-derived corrosion rankings is interpreted in terms of the competition between global passive-film protection and local interfacial activation. The present study is expected to provide both theoretical guidance and experimental support for process optimization and the synergistic improvement of service performance in LMD-fabricated TiC particle-reinforced 316L-based composites.

## 2. Materials and Methods

### 2.1. Raw Materials

**Figure 1:**
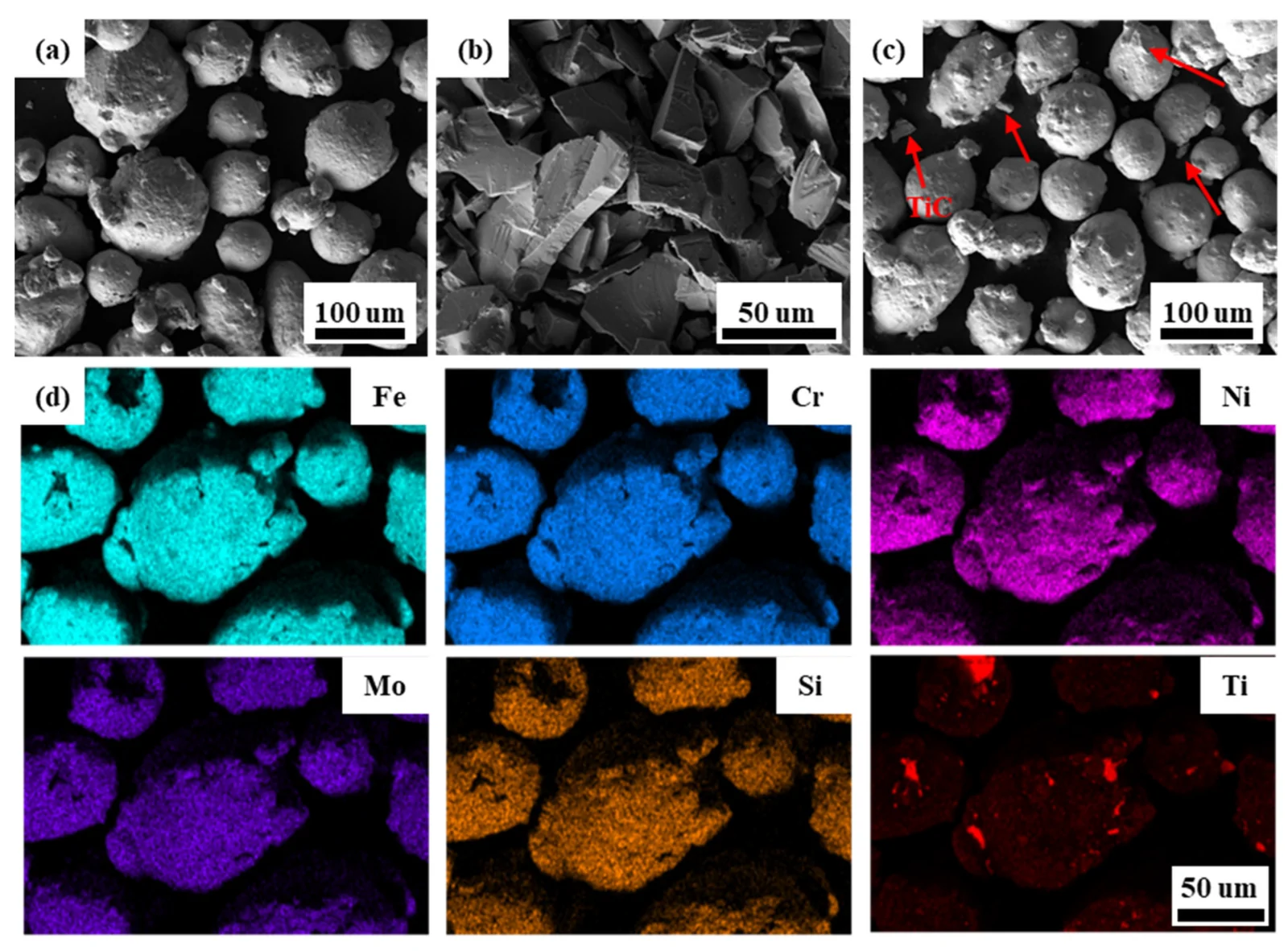
SEM morphologies of raw powders: (**a**) 316L stainless steel powder; (**b**) raw TiC particles; (**c**) mixed 316L/TiC composite powder; (**d**) EDS elemental mapping of the 316L/TiC composite powder.

The feedstock powders used in this work consisted of gas-atomized spherical 316L stainless steel powder and micron-sized TiC particles. According to the experimental design, the particle size of the 316L powder ranged from 53 to 150 μm, the TiC particle size was 15–45 μm, and the mass fraction of TiC was fixed at 5 wt.%. The as-received 316L powder exhibited a nearly spherical morphology (Figure 1a) and favorable powder flowability. The detailed chemical composition of the 316L powder provided by the supplier is listed in Table 1. In contrast, the raw TiC particles displayed irregular, angular block-like shapes (Figure 1b).

To fabricate composite feedstock with homogeneous elemental distribution, the weighed 316L and TiC powders were blended via planetary ball milling. The ball-milling parameters were set as follows: a ball-to-powder mass ratio of 5:1, a rotational speed of 200 r/min, and a continuous milling duration of 2 h. After milling, the mixed powder was transferred to a vacuum drying oven and held at 120 °C for 4 h to completely eliminate residual moisture, yielding well-dispersed composite powder. As illustrated in Figure 1c, spherical 316 particles remained the dominant phase after ball milling, while TiC particles adhered to or were embedded on the surface of 316L spheres, demonstrating that mechanical mixing effectively improved the dispersion of the reinforcing phase.

Scanning electron microscopy (SEM) coupled with energy-dispersive X-ray spectroscopy (EDS) was adopted to characterize the morphologies and elemental distribution of both pristine and mixed powders. EDS mapping results (Figure 1d) revealed that Fe, Cr, Ni, Mo and Si were uniformly distributed within the 316L matrix particles, whereas Ti was concentrated on adhered Ti-rich regions, which verified the successful incorporation of TiC into the 316L powder system. The obtained composite powder was subsequently utilized for LMD experiments to investigate the effects of laser power on the microstructure and corrosion resistance of as-deposited 316L/TiC composite coatings.

Given the micron scale of TiC particles, partial retention of their original morphology can be achieved inside the LMD molten pool; nevertheless, surface melting, partial decomposition and elemental interdiffusion between TiC and the liquid matrix may occur in local high-temperature zones. Accordingly, the homogeneity of powder mixing directly governs the spatial distribution of TiC reinforcements within the deposited layer and further exerts significant influence on the formation of localized defects and the overall electrochemical corrosion performance of the composite coating.

### 2.2. Sample Fabrication

Single-track 316L/TiC composite deposits were fabricated via laser metal deposition (LMD), with 304 austenitic stainless steel selected as the substrate material. In this study, 304 austenitic stainless steel was used as the substrate rather than 316L, mainly because its principal physical properties are comparable to those of 316L, whereas its cost is relatively lower. A 2000 W-rated fiber laser system was employed, and the laser spot diameter was fixed at 3 mm. The standoff distance between the laser cladding nozzle tip and the top surface of the substrate was set to 15 mm. Constant processing parameters were maintained throughout deposition: laser scanning speed of 5 mm/s, high-purity argon shielding gas flow rate of 12 L/min, and powder feeder disc rotational speed of 1.5 rpm. Four groups of specimens were produced by adjusting the laser output power to 1000 W, 1200 W, 1400 W and 1600 W, respectively. The processing parameters used in this study were selected on the basis of our preliminary experimental results. Specifically, the chosen laser power range ensured sufficient powder melting and the formation of a sound, defect-free metallurgical bonding interface; meanwhile, the proper matching between laser power and scanning speed was conducive to obtaining a relatively high single-track cladding layer. For concise labeling, the samples fabricated at 1000 W, 1200 W, 1400 W and 1600 W are abbreviated as P10, P12, P14 and P16 in the subsequent discussion.

### 2.3. Characterization of Samples

After cladding, the substrates were uniformly machined by wire electrical discharge cutting into square specimens with dimensions of 10 mm × 10 mm. The samples were then subjected to sectioning, grinding, polishing, and, when necessary, etching prior to microstructural characterization. Optical microscopy (OM) was used to examine the polishing quality, clad bead morphology, and metallographic features of the samples. Scanning electron microscopy (SEM) was employed to analyze the cellular/dendritic structures, particle distribution, local defects, and interfacial morphology within the deposited layers. Energy-dispersive spectroscopy (EDS) was used to characterize the elemental distributions in the particle-rich regions and matrix regions, with particular attention given to the relative enrichment or depletion of Ti and C as well as Fe, Cr, Ni, and Mo. To further determine whether TiC particles were fully retained and to clarify the phase constitution, especially the relative proportions of austenite and martensite, X-ray diffraction (XRD) analysis was conducted. The XRD measurements were performed over a 2θ range of 30–90° at a scanning rate of 4°/min to identify the phase composition and crystal structure of the clad layers.

The microhardness of the coatings was measured along the thickness direction using a high-precision Vickers microhardness tester. The spacing between adjacent indentations was 300 μm, the applied load was 300 g, and the dwell time was 10 s. To reduce measurement uncertainty, three hardness values were collected at each testing position at the same height on the cross-section, and the average value was reported.

Room-temperature dry sliding wear tests were carried out using a reciprocating tribometer to evaluate the tribological performance of the coatings, while the average coefficient of friction (COF) was recorded throughout the entire test. A silicon nitride (Si_3_N_4_) ceramic ball with a diameter of 4 mm was used as the counterface material. The normal load was set to 15 N, the reciprocating frequency was 2 Hz, the sliding stroke for each cycle was 5 mm, and the total test duration was 30 min. All wear tests were repeated at least three times to ensure the reliability and reproducibility of the results. After testing, the dimensions and morphology of the wear tracks were measured using a Bruker Contour GT-K1 three-dimensional surface profilometer (Bruker Nano Inc., Billerica, MA, USA).

The corrosion resistance of the coatings was evaluated by electrochemical measurements using an electrochemical workstation, from which Tafel polarization curves and electrochemical impedance spectroscopy (EIS) data were obtained for corrosion analysis. The Tafel curves were used to compare the polarization behavior, corrosion potential, and current response of different samples, whereas the EIS spectra were employed to evaluate the impedance response and interfacial corrosion processes. A conventional three-electrode cell was adopted to assess the corrosion behavior of the coatings at room temperature in a 3.5 wt.% NaCl solution simulating seawater. The coated samples were cold-mounted, successively ground and polished, and then used as the working electrodes. A saturated calomel electrode (SCE) and a platinum plate served as the reference and counter electrodes, respectively. Prior to electrochemical testing, the open-circuit potential (OCP) was monitored for 30 min until a relatively stable state was reached. Subsequently, non-destructive EIS measurements were performed. The AC perturbation amplitude was set to 10 mV, and the frequency range was from 10^−2^ to 10^6^ Hz. The impedance data were fitted using the Gamry supporting software Installation Version 7.8.2.7430 to establish the corresponding equivalent circuit model. Finally, potentiodynamic polarization tests were conducted over a potential range of −1.5 to 1.5 V at a scanning rate of 1 mV/s^−1^, and the resulting data were used to analyze the corrosion kinetic characteristics of the coatings.

## 3. Results and Discussion

### 3.1. Microstructure and Distribution of TiC Particles

**Figure 2:**
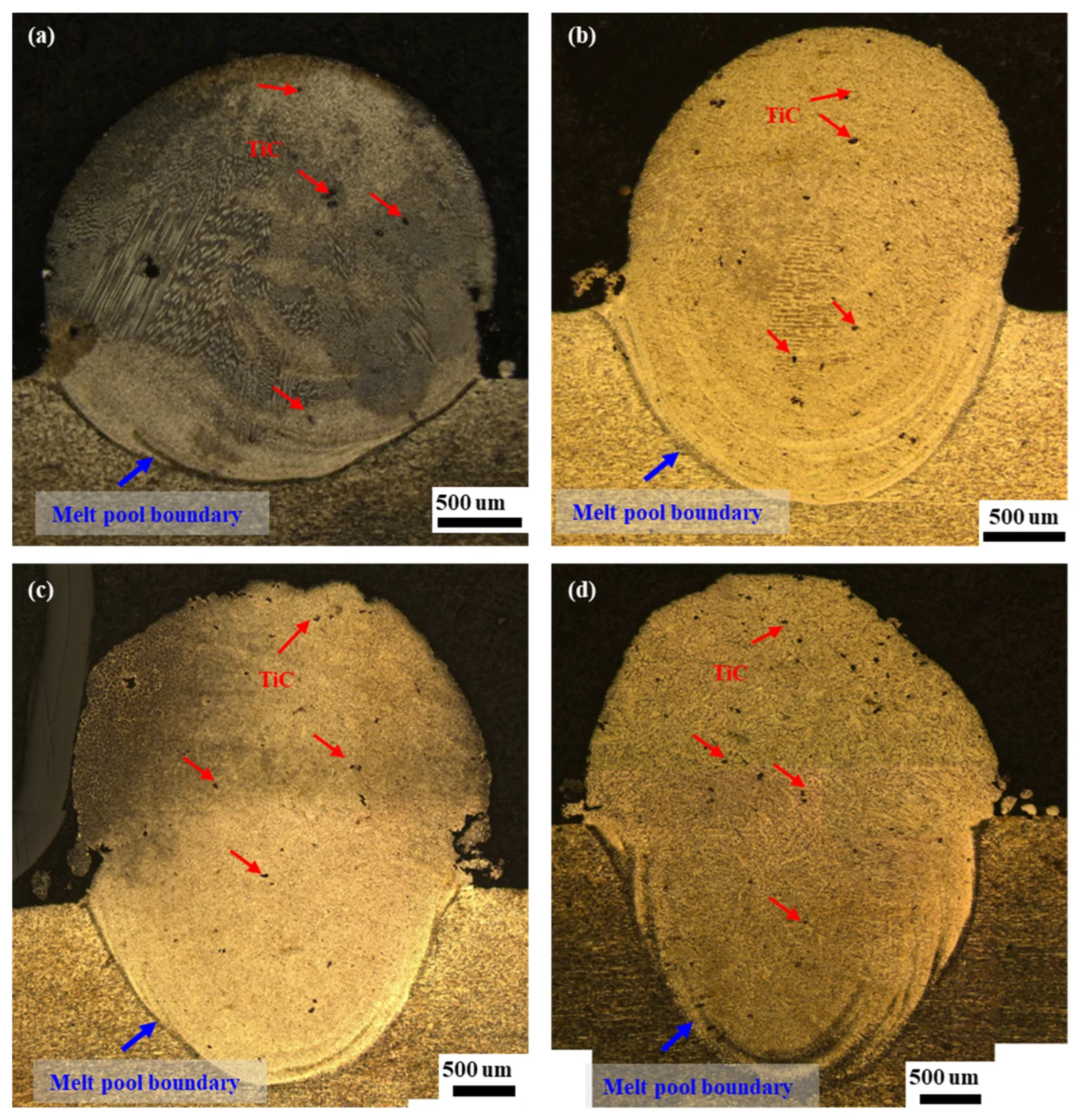
Cross-sectional morphologies of 316L/TiC composite coatings: (**a**) P10; (**b**) P12; (**c**) P14; (**d**) P16.

As shown in Figure 2, all 5.0 wt.% TiC/316L composite coatings prepared at different laser powers exhibit good formability and continuous coating/substrate interfaces, indicating good metallurgical bonding. The cross-sections are generally semi-elliptical, but their geometries vary systematically with laser power, i.e., with the corresponding linear energy density E = P/V. At a constant scanning speed of 5 mm/s, the energy densities for P10, P12, P14, and P16 are 200, 240, 280, and 320 J/mm, respectively.

At the lowest energy density (P10, 200 J/mm), the coating shows a relatively “tall and narrow” morphology with limited penetration into the substrate, suggesting a relatively low dilution ratio. With increasing energy density to 240 J/mm (P12), the clad track becomes fuller and more regular, implying a more stable molten pool and improved deposition behavior. When the energy density further increases to 280 and 320 J/mm (P14 and P16), the penetration depth becomes more pronounced and the melt-pool boundary at the bottom is clearer, indicating enhanced substrate melting and a higher dilution ratio.

These results demonstrate that increasing heat input enlarges the molten pool, especially in the depth direction, and gradually increases substrate participation in the cladding process. A moderate dilution effect is beneficial for metallurgical bonding, whereas excessive dilution may decrease the relative fraction of externally added TiC particles and modify the melt composition and solidification conditions, thereby affecting the microstructural evolution and particle distribution in the coating.

**Figure 3:**
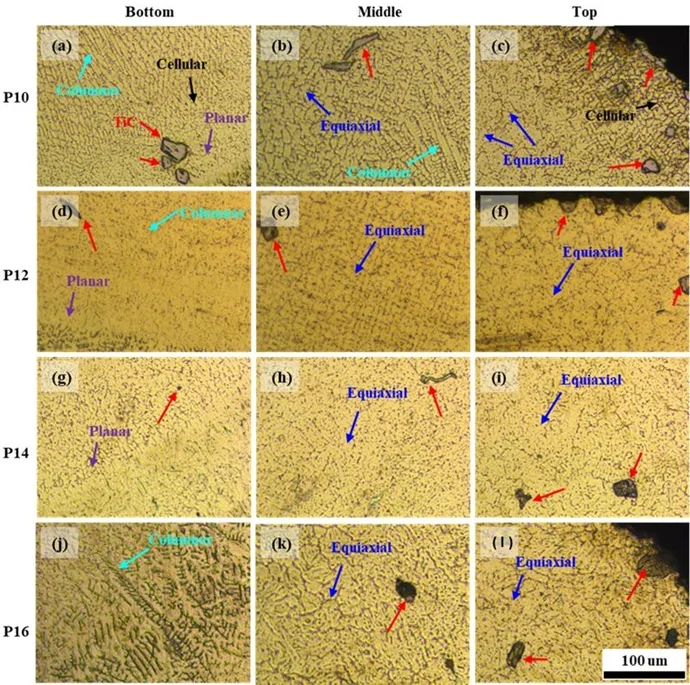
Microstructures at the bottom, middle and top regions of cross-sections for 316L/TiC composite coatings: (**a**–**c**) are the microstructures of the bottom, middle, and top of the P10 sample, respectively. (**d**–**f**) are the microstructures of the bottom, middle, and top of the P12 sample, respectively. (**g**–**i**) are the microstructures of the bottom, middle, and top of the P14 sample, respectively. (**j**–**l**) are the microstructures of the bottom, middle, and top of the P16 sample, respectively.

Figure 3 shows that all composite coatings were predominantly composed of austenitic structures, together with a small amount of undissolved TiC particles, which is are consistent with previous reports [23,27]. Along the thickness direction, the microstructure generally evolved from planar/cellular crystals and columnar grains near the bottom to equiaxed grains in the middle and upper regions. This transition is mainly associated with variations in the temperature gradient (G) and crystal growth rate (R) during the rapid solidification process of laser cladding [28,29]. Near the fusion line, rapid heat dissipation and a relatively high G/R ratio favor the formation of planar and columnar crystals. With increasing distance from the substrate, the temperature gradient decreases and constitutional undercooling becomes more pronounced, thereby promoting the nucleation and growth of equiaxed grains [26,30]. A small fraction of undissolved TiC particles was dispersed within the coating, some of which were located in the middle and upper regions. Owing to their high melting point, these particles were not fully melted in the melt pool and could act as heterogeneous nucleation sites for austenite, thereby reducing the nucleation barrier, refining the microstructure, and hindering grain boundary migration [26]. Overall, laser power significantly affected the forming characteristics and microstructural evolution of the composite coatings by altering the thermal behavior of the melt pool, the dilution ratio, and the distribution state of TiC particles.

**Figure 4:**
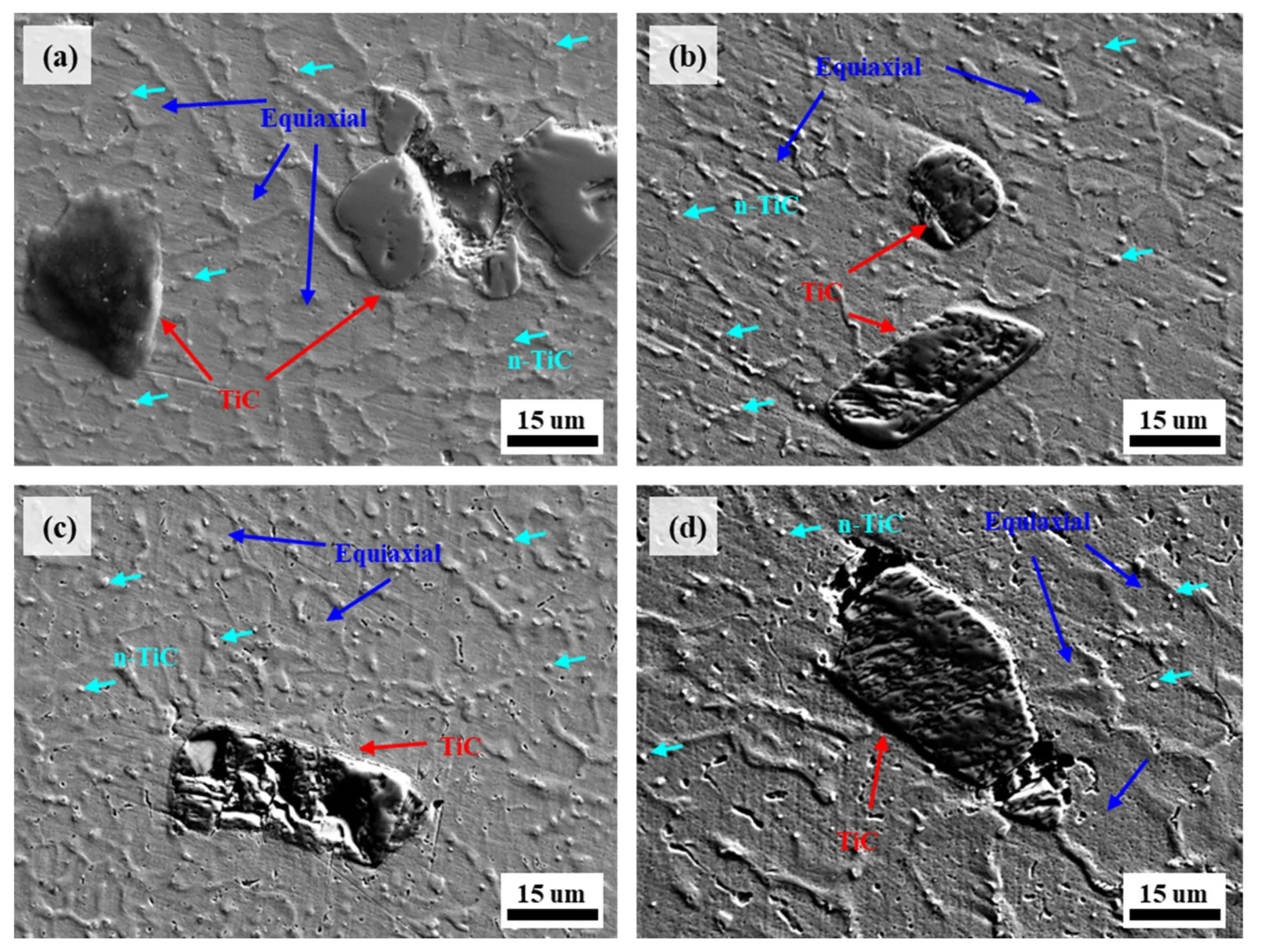
Micrographs at the middle cross-section of composite coatings: (**a**) P10; (**b**) P12; (**c**) P14; (**d**) P16.

As shown in Figure 4, in addition to a small amount of undissolved TiC particles, a large number of dispersed nanoscale particles were also observed in the composite coatings. This phenomenon is closely related to the high energy density, short interaction time, and rapid solidification characteristics of the laser melt deposition process. TiC is a high-melting-point ceramic phase with a melting point of approximately 3140 °C, which is much higher than the melting temperature of the 316L stainless steel matrix. Therefore, under conditions of limited laser interaction time and a non-uniform temperature field within the melt pool, some relatively large TiC particles or particles located in locally low-temperature regions are difficult to melt completely and only undergo edge erosion or partial dissolution, finally remaining in the coating as undissolved particles [21,31].

Meanwhile, the high temperature of the melt pool can also cause partial dissolution of some TiC particles, particularly at their surfaces, thereby releasing Ti and C atoms into the liquid phase and forming locally Ti-rich and C-rich melts. As the laser beam moves away, the melt pool rapidly solidifies under a high cooling rate. The solubility of Ti and C in austenite then decreases sharply, resulting in pronounced supersaturation of the system and the reprecipitation of TiC-rich particles under the driving force of significant constitutional undercooling [21,22]. Because of the extremely rapid cooling and limited diffusion time during laser cladding, the reprecipitated phase has insufficient time to grow substantially and therefore mainly exists in the form of dispersed nanoparticles [26,31]. On the other hand, the surfaces of undissolved TiC particles can also serve as heterogeneous nucleation substrates, lowering the nucleation barrier for reprecipitated TiC-rich particles and promoting their preferential nucleation in the vicinity of these particles [31]. As a result, a composite structure consisting of “undissolved TiC particles + reprecipitated TiC-rich particles” is formed in the coating, in which the former mainly acts as a hard reinforcing phase, whereas the latter improves the microstructure and properties through dispersion strengthening and grain refinement. Since the reprecipitated features observed in this study are extremely fine and uniformly dispersed, and more importantly, do not exhibit the sharp angular morphology typically associated with fragmented TiC particles, the possibility that they originated merely from the mechanical breakage of larger TiC particles reported in previous studies can be reasonably excluded [23].

Variations in laser power further determine the relative proportion and distribution characteristics of these two types of TiC particles. At lower laser power, the heat input is insufficient, resulting in a lower melt-pool temperature and a shorter high-temperature dwell of the molten pool; consequently, the extent of TiC dissolution is limited. As a consequence, more undissolved TiC particles are retained in the coating, while the amount of reprecipitated TiC-rich particles is relatively low. With increasing laser power, the heat input into the melt pool rises, enhancing heat transfer and interfacial reactions between TiC particles and the melt pool. Consequently, more TiC dissolves, providing a sufficient solute source for the precipitation of TiC-rich particles during subsequent rapid solidification. As a result, the amount of nanoscale precipitates increases, and their distribution becomes more uniform. However, if the laser power is excessively high, melt-pool overheating and prolonged residence time will intensify TiC dissolution and convective migration of elements and may also promote the coarsening of already precipitated particles or even lead to reinforcement segregation and microstructural inhomogeneity. Therefore, a moderate laser power is more favorable for forming a multiscale reinforcing structure characterized by the synergistic coexistence of undissolved TiC and reprecipitated TiC-rich particles, thereby balancing particle retention, dispersion strengthening, and microstructural uniformity. Although previous investigations [26] have reported that increasing volumetric energy density accelerates the dual coarsening pathways of TiC particles, i.e., nanoscale TiC grows via atomic diffusion while microscale TiC coarsens through collision and coalescence within the molten pool, such conclusions cannot be directly extrapolated to the present work. This discrepancy arises from fundamental differences in experimental conditions: the prior research adopted low-power laser powder bed fusion (LPBF) and employed initial TiC particles with a limited average size of 5 μm, whereas the current study utilizes laser metal deposition (LMD) and much coarser raw TiC particles up to 45 μm in diameter.

### 3.2. Phase Analysis of the 316L/TiC Composite Coatings

**Figure 5:**
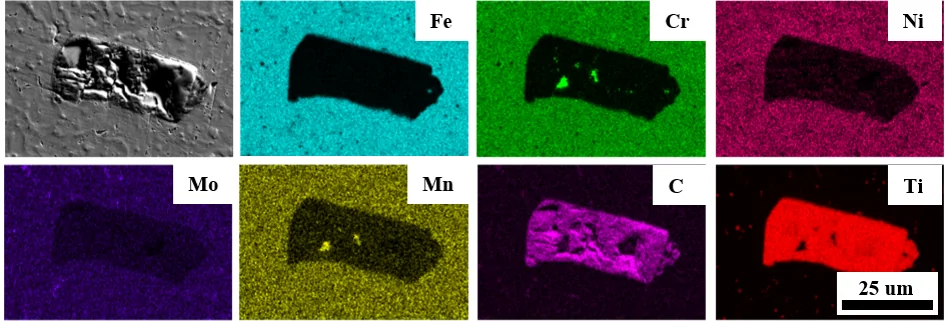
EDS mappings of undissolved TiC particles in the middle cross-section of the P16 composite coating.

**Figure 6:**
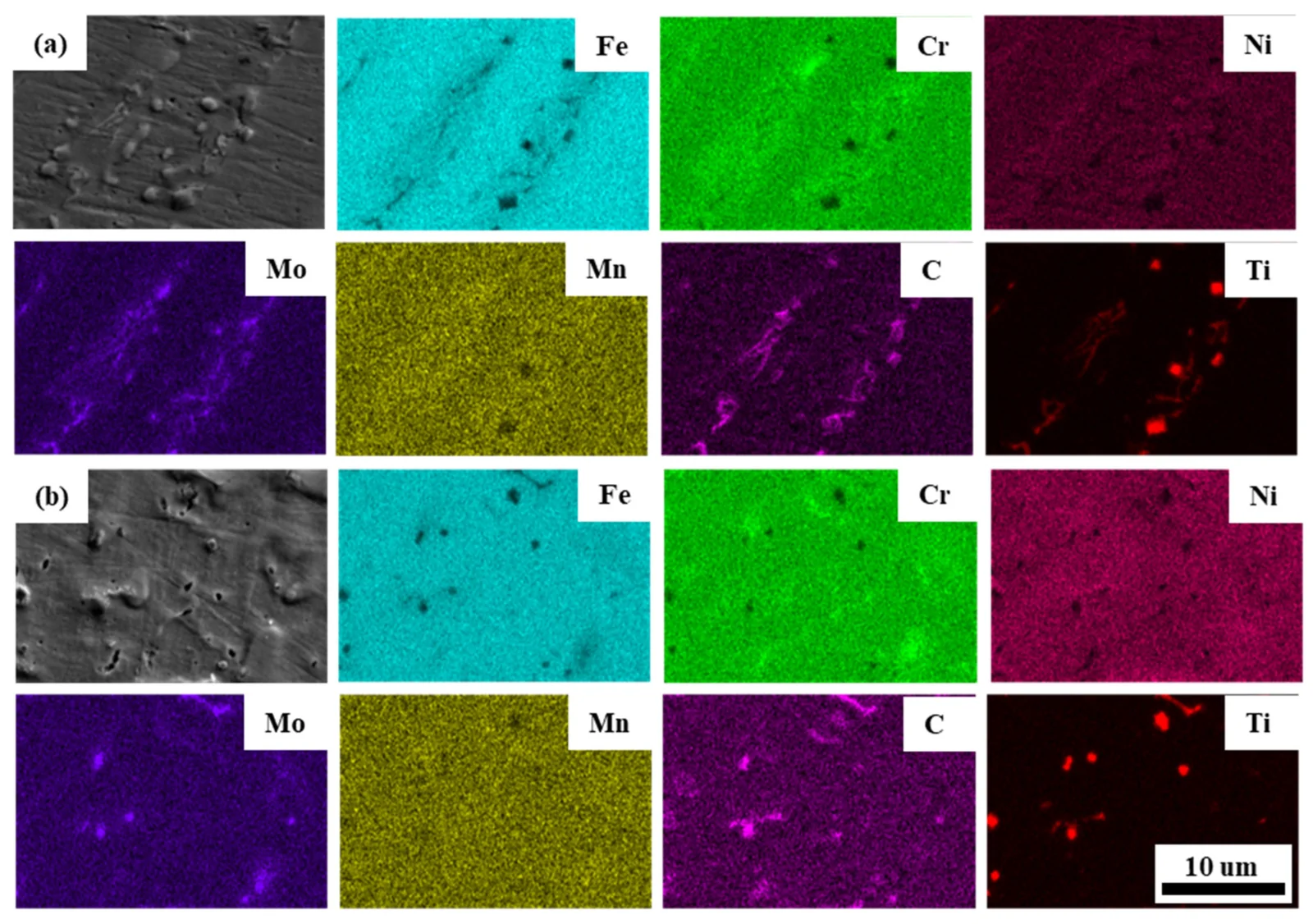
EDS spectra of reprecipitated TiC-rich particles in the middle cross-section of composite coatings: (**a**) Specimen P10; (**b**) Specimen P16.

The EDS mapping results shown in Figure 5 and Figure 6 further confirm that the 316L/TiC composite coatings are composed of an austenitic matrix, undissolved TiC particles, and reprecipitated nanoscale TiC-rich particles. For representative compositional characterization, the P16 specimen was selected for EDS mapping of the retained undissolved TiC particles. As the sample fabricated under the highest laser power, P16 exhibited more distinct particle/matrix interfacial features and local compositional heterogeneity, which facilitated clearer observation of the elemental distribution in the particle-containing region. For the coarse blocky particles located in the middle region of the cross-section, Ti and C are obviously enriched, whereas the signals of matrix elements such as Fe, Cr, Ni, Mo, and Mn are markedly weakened, indicating that these particles are primarily undissolved or partially dissolved TiC particles (Figure 5).

Based on the local Ti/C enrichment (Figure 6), microstructural morphology, and consistency with previous reports on TiC-reinforced laser-clad materials, the fine dispersed features are reasonably inferred to be TiC-rich reprecipitates formed during rapid solidification after partial dissolution of TiC particles [20,21]. EDS results also indicate that, in addition to the undissolved TiC particles, the reprecipitated nanoscale TiC-rich regions in the coatings are enriched not only in Ti and C, but also in Mo (Figure 6). This suggests that the formation of TiC-rich particles is not simply a consequence of the reprecipitation of locally dissolved TiC, but is also closely related to solute redistribution during rapid solidification. During the laser melting deposition process, some TiC particles undergo partial dissolution in the high-temperature molten pool, releasing Ti and C atoms, which subsequently reprecipitate as TiC-rich particles in the vicinity of the original particles or within interdendritic regions during rapid solidification. Meanwhile, owing to its relatively low diffusion rate and pronounced tendency for interfacial segregation, Mo tends to accumulate in interdendritic residual liquid regions, solute-enriched zones, and near particle/matrix interfaces. In addition, the TiC-rich particle formation regions are generally associated with relatively high interfacial energy and localized stress fields, which further promote Mo enrichment in these areas.

**Figure 7:**
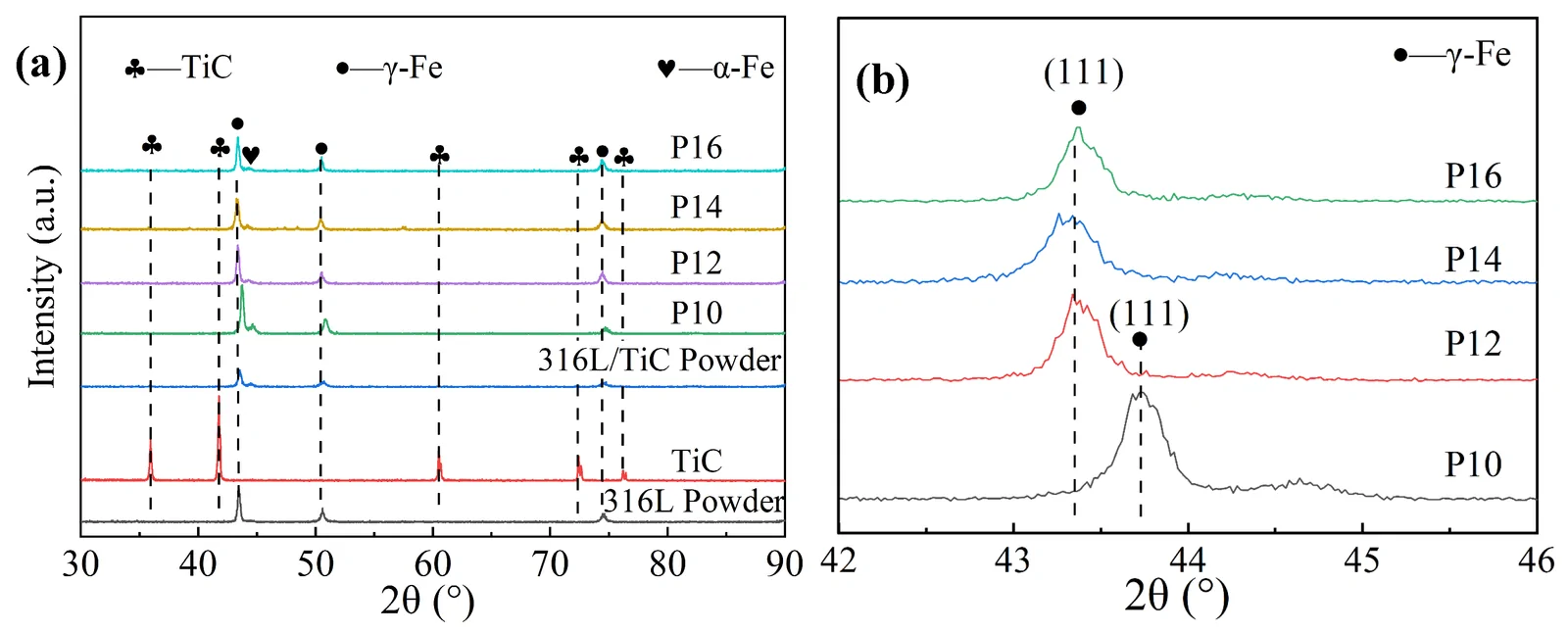
XRD patterns of raw powder and composite coatings fabricated under various laser powers: (**a**) global spectra at 30–90°; (**b**) 42–46° local magnification.

Figure 7a shows that the as-received 316L powder mainly consists of the γ-Fe phase, while the TiC powder exhibits distinct TiC characteristic peaks. Correspondingly, both γ-Fe and TiC diffraction peaks are present in the mixed powder. After cladding, the 316L/TiC composite coatings fabricated at different laser powers are all dominated by the γ-Fe phase, accompanied by relatively weak TiC diffraction peaks and a faint α-Fe peak. This indicates that the coating matrix remains predominantly austenitic while retaining part of the TiC reinforcing phase, which is consistent with the microstructural observations and EDS results. The dominance of the γ-Fe phase is mainly attributed to the austenite-stabilizing effects of Ni and Cr in 316L, together with the suppression of diffusion-controlled phase transformations under rapid laser solidification. The presence of TiC peaks indicates that, owing to the high melting point of TiC, the short laser interaction time, and the non-uniform temperature field in the melt pool, some TiC particles are not completely dissolved and are retained in the coating in the form of residual particles and/or reprecipitated phases [18]. The appearance of a weak α-Fe peak may be related to substrate dilution, local compositional segregation, and the formation of a small amount of ferrite under non-equilibrium solidification conditions during laser cladding [4,32].

With increasing laser power, the heat input to the melt pool rises, which enhances the edge erosion and local dissolution of TiC particles. As a result, part of the Ti and C atoms enter the austenitic matrix and subsequently reprecipitate as fine TiC particles during rapid solidification. Because the reprecipitated phase is very fine and its content is limited, its XRD response is relatively weak. Therefore, the TiC peaks in the diffraction patterns mainly represent the combined contribution of residual coarse TiC particles and part of the reprecipitated TiC. As shown in Figure 7b, the γ-Fe (111) peak exhibits a certain shift with laser power, indicating a change in the lattice parameter of austenite. This can mainly be attributed to the following factors: partial solid solution of Ti and C atoms into the γ-Fe matrix, resulting in lattice distortion; changes in interplanar spacing caused by residual stresses induced by rapid laser heating and cooling [22]; and peak shift and broadening associated with grain refinement and increased microstrain [33,34]. In addition, as laser power increases, enhanced matrix melting and a higher dilution ratio lead to changes in the actual composition of the coating, which may further affect the lattice constant of γ-Fe [18]. Therefore, by regulating the retention, dissolution, and reprecipitation behavior of TiC, as well as the matrix composition and residual stress state, laser power significantly influences the phase constitution and diffraction characteristics of the composite coatings.

### 3.3. Microhardness of the 316L/TiC Composite Coatings

**Figure 8:**
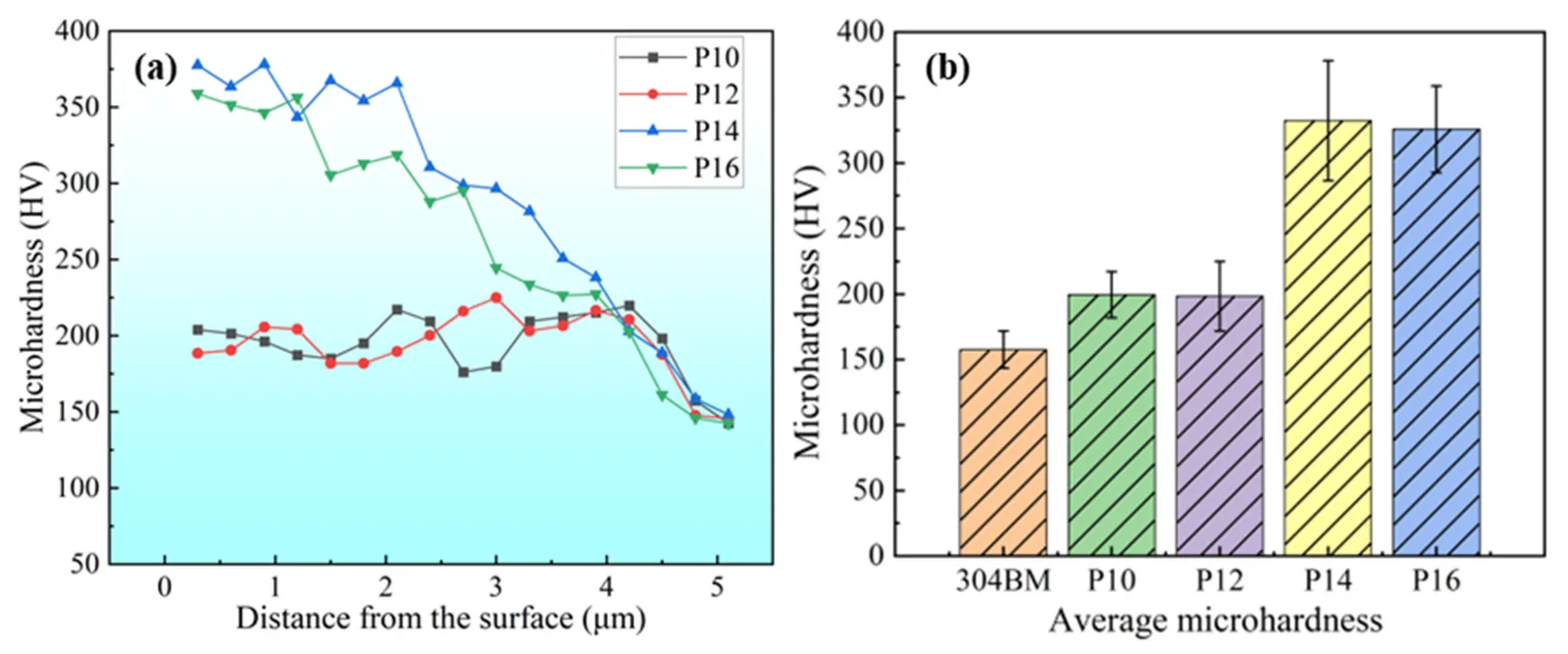
Microhardness results of 316L/TiC composite coatings prepared with different laser powers: (**a**) Hardness distribution from coating surface to substrate; (**b**) Average microhardness values of coatings at distinct laser powers.

Figure 8 shows that the microhardness values of the 316L/TiC composite coatings fabricated under different laser powers are all markedly higher than those of the 304BM substrate, indicating that the introduction of TiC particles together with the rapid solidification inherent to laser processing can significantly improve the resistance of the coatings to plastic deformation. The average hardness follows the order of P14 ≈ P16 > P12 ≈ P10 > 304BM. Specifically, the average hardness of P14 and P16 is approximately 320–330 HV, whereas that of P10 and P12 is only around 200 HV (Figure 8b). Combined with the microstructural analysis, it can be seen that the coatings mainly consist of an austenitic matrix, a small amount of undissolved TiC particles, and a large number of reprecipitated nanoscale TiC particles. The increase in coating hardness is mainly attributed to the dispersion-strengthening effect of TiC particles and their grain-refinement effect on the austenitic matrix [30,35]. At relatively low laser powers (P10 and P12), the heat input to the melt pool is insufficient, the dissolution degree of TiC particles is limited, and the amount of reprecipitated nanoscale TiC is relatively low; consequently, the dispersion-strengthening effect is weak, and the increase in hardness is limited. As the laser power increases to P14, the melt-pool temperature rises and mass transfer is enhanced, enabling more TiC to undergo local dissolution and subsequently precipitate as a large number of fine, dispersed nanoscale TiC particles during rapid solidification. At the same time, the microstructure is refined, giving rise to pronounced fine-grain strengthening and dispersion strengthening, and thus the coating hardness reaches its maximum. Although the P16 coating still maintains a relatively high hardness, the local fluctuation becomes more pronounced, suggesting that excessive heat input, while favorable for TiC dissolution and reprecipitation, may also induce local grain coarsening or a non-uniform distribution of the reinforcing phases. In addition, it can be seen from Figure 8a that the hardness of all cladding layers generally decreases from the top surface toward the substrate. This is mainly because the surface region of the clad layer experiences a higher cooling rate and therefore develops a finer microstructure, while the strengthening effect of TiC particles is more pronounced in this region. By contrast, near the fusion line, the enhanced dilution effect from the substrate weakens both the content of the reinforcing phases and the microstructural refinement effect, leading to a reduction in hardness.

### 3.4. Wear Behavior of the 316L/TiC Composite Coatings

**Figure 9:**
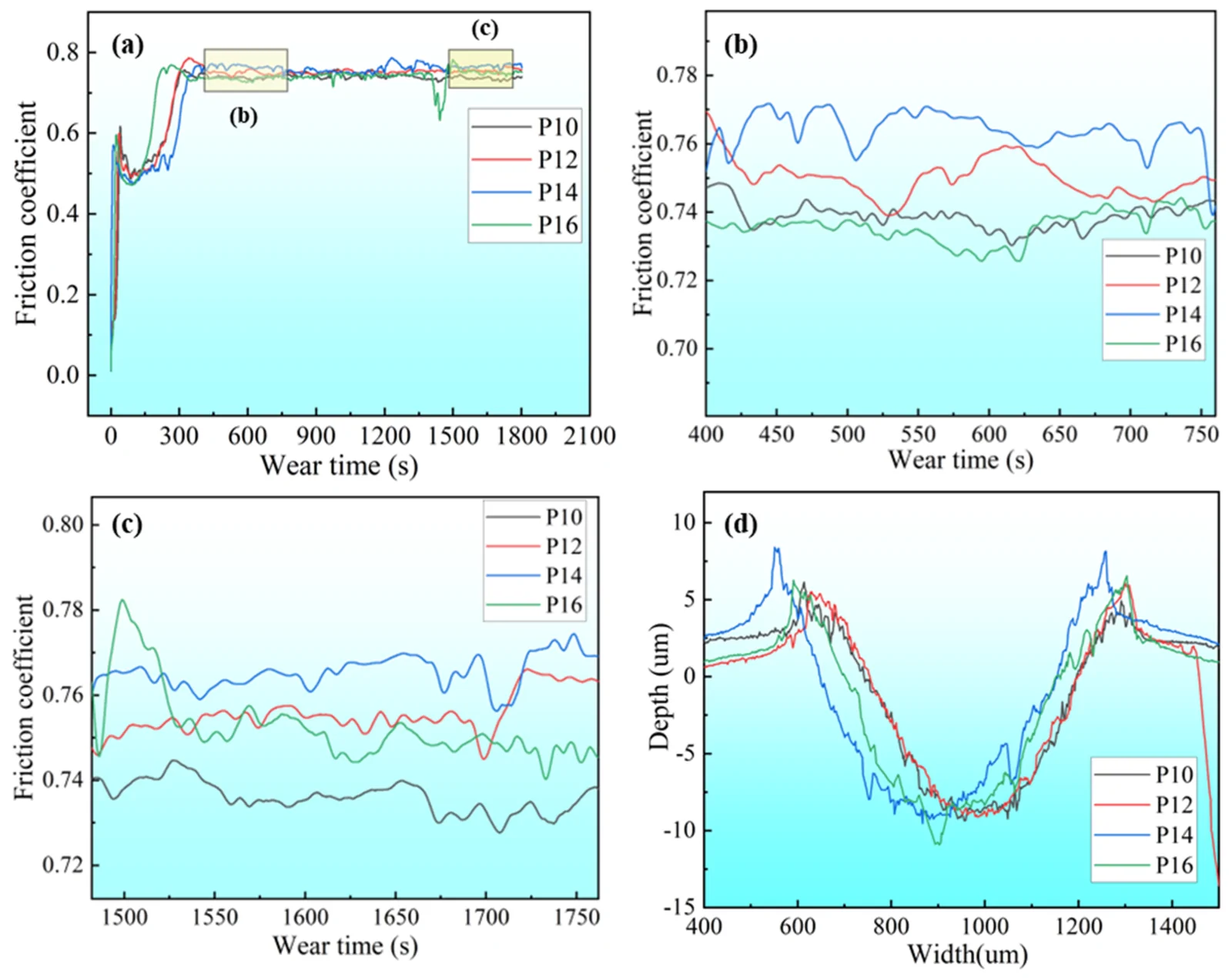
Friction coefficient curves and cross-sectional wear track profiles of 316L/TiC composite coatings under different laser powers: (**a**–**c**) Friction coefficient plots; (**d**) Cross-sectional wear track profile.

**Figure 10:**
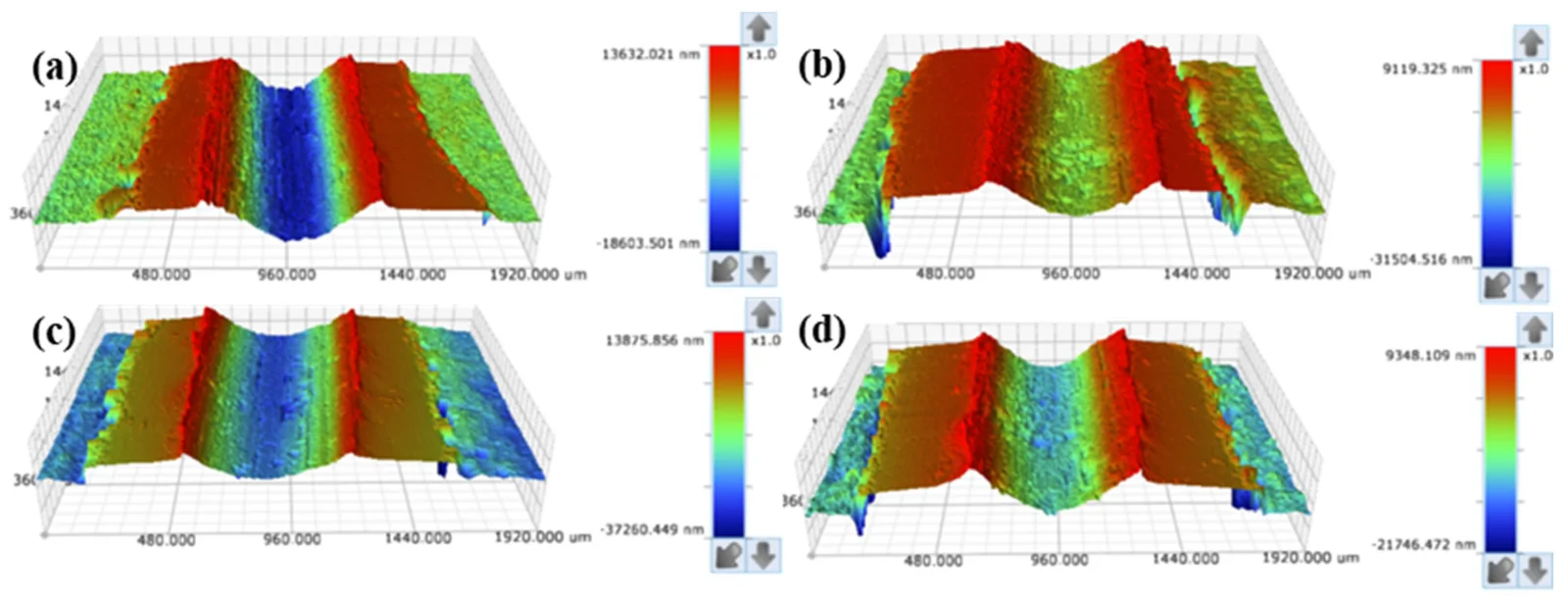
Three-dimensional wear track morphologies of 316L/TiC composite coatings with varying laser powers: (**a**) P10; (**b**) P12; (**c**) P14; (**d**) P16.

Figure 9 and Figure 10 show the room-temperature friction and wear behavior of the 5.0 wt.% TiC/316L composite coatings prepared at different laser powers. As shown in Figure 9, all coatings exhibit a transition from the initial running-in stage to a relatively stable wear stage. During steady-state sliding, the friction coefficient varies within 0.73–0.77, with P10 showing the lowest value (0.73) and P14 the highest (0.77), while P12 (0.76) and P16 (0.75) lie in between. Although the difference in friction coefficient is limited, the wear-track profiles reveal clearer distinctions in actual wear damage.

For quantitative evaluation, the cross-sectional profiles in Figure 9d are more reliable than the color-rendered three-dimensional morphologies in Figure 10. The measured wear-track depths are 15.09 μm (P10), 16.55 μm (P12), 17.59 μm (P14), and 16.06 μm (P16), and the corresponding widths are 659.97, 678.29, 698.91, and 674.14 μm, respectively. These results indicate that P10 has the shallowest and narrowest wear scar, whereas P14 shows the deepest and widest track. P12 and P16 exhibit intermediate wear damage with only small differences between them.

Combined with the wear morphology in Figure 10, the results suggest that P10 undergoes the least material removal and thus has the best wear resistance, while P14 suffers the most severe ploughing and local spallation, resulting in the poorest wear resistance. Therefore, the overall wear resistance can still be ranked as P10 > P16 ≈ P12 > P14, indicating that wear performance is governed not only by hardness, but also by microstructural uniformity, reinforcing-phase distribution, and interfacial stability.

The wear resistance of the composite coatings depends not only on hardness, but also strongly on microstructural uniformity, the distribution of TiC particles, and the interfacial bonding state between the particles and the matrix [24,33]. At relatively low laser powers, the cladding layer exhibits a more uniform microstructure and a more reasonable particle distribution. During friction, the tendency for particle pull-out and local delamination is relatively weak. A small amount of undissolved TiC particles, serving as high-hardness reinforcing phases, can enhance the resistance of the austenitic matrix to plastic deformation and micro-cutting, thereby improving wear resistance. As a result, P10 exhibits the best wear performance. However, wear resistance is not determined solely by hardness. As the laser power increases, although the hardness rises further, local microstructural heterogeneity, interfacial stress concentration, and the tendency for particle detachment may also increase. Detached TiC particles can then act as third-body abrasives during sliding, thereby aggravating ploughing and groove wear, which leads to a marked deterioration in the wear resistance of the P14 sample. Under the higher heat input condition, the wear resistance of P16 is somewhat improved relative to that of P14, but it is still inferior to that of P10. This indicates that, for the 5.0 wt.% TiC/316L composite coatings, a moderate laser power is more favorable for balancing hardness enhancement and wear-performance optimization.

### 3.5. EIS Analysis of the 316L/TiC Composite Coatings

**Figure 11:**
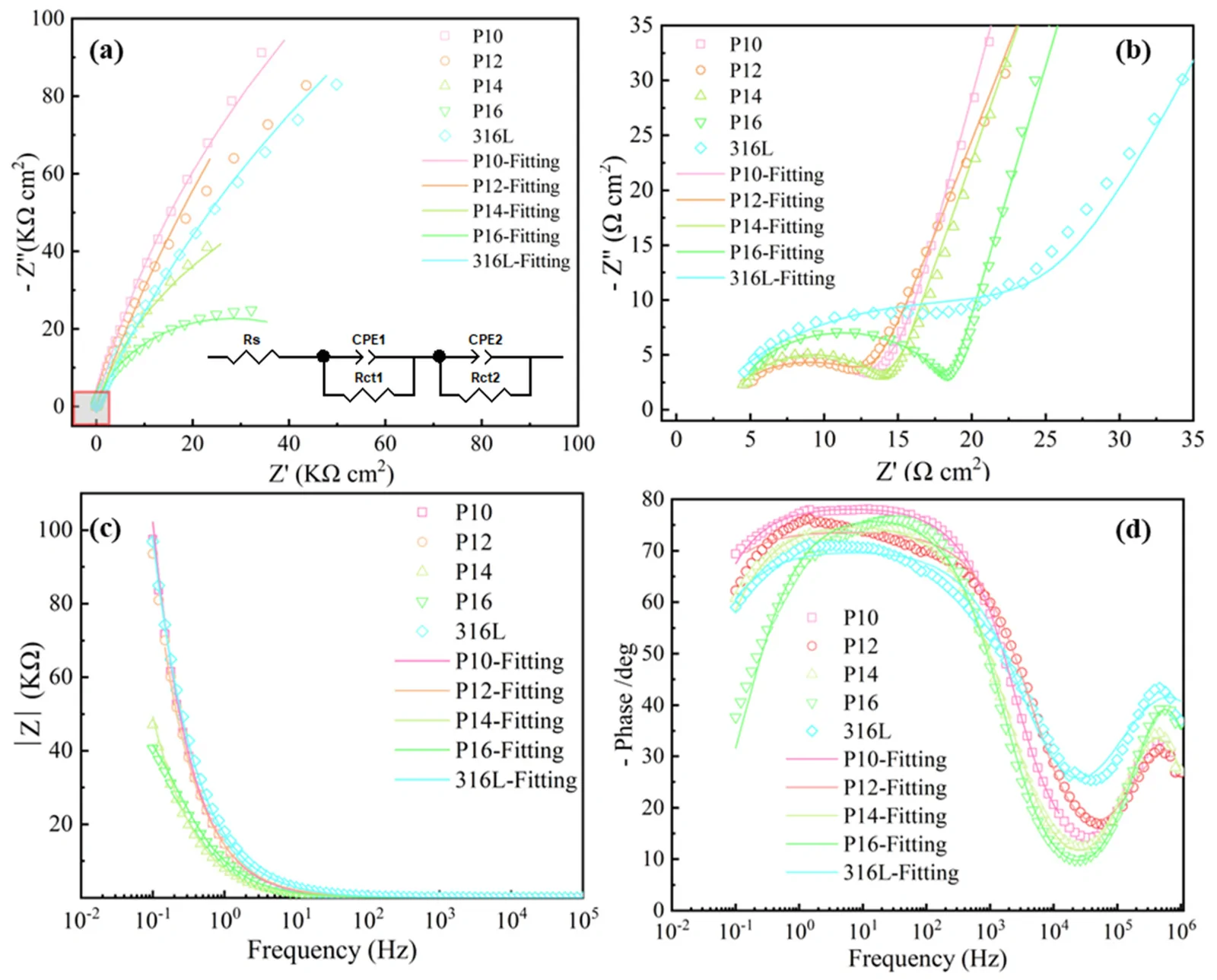
EIS of 316L/TiC composite coatings fabricated at different laser powers: (**a**) Nyquist plots; (**b**) Magnified high-frequency region of Nyquist plots; (**c**) Bode magnitude plots; (**d**) Bode phase plots.

To further evaluate the electrochemical behavior of the 5.0 wt.% TiC/316L cladding layers in a corrosive medium under different laser powers, EIS measurements were performed on all samples. The results are presented in Figure 11, and the corresponding fitting parameters are listed in Table 2. The impedance spectra of all samples were fitted using a two-time-constant equivalent circuit, $R_{s}-({CPE}_{1}∥R_{ct1})-({CPE}_{2}∥R_{ct2})$, where $R_{s}$ is the solution resistance; $R_{ct1}$ and ${CPE}_{1}$ mainly represent the electrochemical response of the surface film and/or the heterogeneous near-surface region; and $R_{ct2}$ and ${CPE}_{2}$ correspond to the charge-transfer behavior at the metal/electrolyte interface [33,36]. The fitted $\chi^{2}$ values for all samples are on the order of $10^{-3}$ to $10^{-4}$, indicating that the selected equivalent circuit describes the impedance characteristics of this system satisfactorily [15,37]. The constant phase element (CPE) was used to account for the non-ideal capacitive behavior arising from film inhomogeneity and adsorption effects [38].

As can be seen from the Nyquist plots, all samples exhibit depressed capacitive semicircles to some extent, indicating pronounced non-ideal capacitive behavior at the clad-layer surface. This is mainly related to the microstructural heterogeneity of the laser-clad layers and the interfacial dispersion effect introduced by a small amount of undissolved TiC particles. Meanwhile, all samples show a certain inflection or a small arc in the high-frequency region (Figure 11b), suggesting the existence of at least two electrochemical relaxation processes in the system, which is consistent with the dual-peak feature observed in the Bode phase-angle plots (Figure 11d). In general, the high-frequency time constant is mainly associated with the response of the passive film, corrosion product film, or the particle/matrix interfacial region, whereas the medium- to low-frequency time constant mainly corresponds to the charge-transfer process at the coating/electrolyte interface [36,39]. Among the samples, P10 and P12 exhibit more regular high-frequency bending features, indicating a more stable surface response, whereas P16 shows more pronounced irregularity, suggesting poorer integrity and uniformity of the surface film.

As shown in Figure 11a, the radii of the Nyquist capacitive arcs vary significantly among the samples. The overall arc radius is the largest for P12, followed by P10 and 316L, then P14, and is the smallest for P16. Generally, a larger Nyquist arc radius indicates a higher charge-transfer resistance and a lower tendency for the corrosion reaction to proceed, and thus better corrosion resistance. Accordingly, it can be preliminarily inferred that P12 exhibits the best corrosion resistance, whereas P16 shows the poorest corrosion resistance.

The Bode modulus plots further confirm this trend. As shown in Figure 11c, significant differences in the impedance modulus ∣Z∣ are observed among the samples in the low-frequency region. Among them, P12 maintains the highest impedance value at the low-frequency end, P10 is close to 316L, P14 decreases markedly, and P16 shows the lowest value. The low-frequency impedance modulus is generally considered to more realistically reflect the overall resistance of a material to corrosive media; the higher its value, the more effectively the interfacial electrochemical reaction is suppressed [16,25]. Therefore, from the perspective of low-frequency ∣Z∣, P12 exhibits the best corrosion resistance, whereas P16 provides the weakest barrier effect against the corrosive medium. In the medium- and high-frequency regions, ∣Z∣ decreases rapidly, corresponding to capacitive behavior and film response. If the surface film is denser and the surface state is more uniform, the frequency-dispersion behavior of the impedance is usually smoother, and a relatively high impedance level can be maintained during the transition toward lower frequencies. In this respect, P12 and P10 exhibit more stable behavior, indicating a more intact near-surface structure. By contrast, the faster decay of P16 suggests a weaker barrier effect of the film and/or surface layer.

The Bode phase-angle plots are shown in Figure 11d. All samples exhibit two phase-angle peaks within the tested frequency range, further confirming the existence of two time constants in the system [33]. P10 and P12 maintain relatively high phase-angle values over a broader frequency range, indicating that their surface films are more stable and that the interfacial capacitive behavior is more pronounced, making it more difficult for the corrosive medium to rapidly penetrate the surface region of the coating and reach the substrate interface [33]. In contrast, P14 and P16 exhibit reduced phase-angle peak values and a narrower high-phase-angle range, indicating weakened protection by the surface film, increased interfacial heterogeneity, and a greater tendency for corrosion to occur. Although the 316L sample also shows relatively high phase angles within part of the frequency range, its overall protective capability is still slightly inferior to that of P12 when considering the full frequency-response characteristics.

Among the fitted parameters, the low-frequency charge-transfer resistance $R_{ct2}$ is an important indicator for evaluating corrosion resistance. As listed in Table 3, the $R_{ct2}$ values of P10, P12, P14, P16, and 316L are $5.33\times 10^{5}$, $6.12\times 10^{5}$, $1.61\times 10^{5}$, $0.56\times 10^{5}$, and $5.55\times 10^{5}\;\Omega \cdot {\mathrm{cm}}^{2}$, respectively. Among these, P12 exhibits the highest $R_{ct2}$, indicating that the interfacial charge-transfer process is most strongly inhibited and that the corrosion reaction is least likely to occur. 316L and P10 rank next, with similar values; P14 decreases markedly; and P16 shows the lowest value, indicating the highest susceptibility to interfacial corrosion reactions. Based on these results, the corrosion resistance of the samples can be ranked as follows: P12 is the best, P10 is close to 316L, followed by P14, and P16 is the worst. It should be noted that the high-frequency branch resistance $R_{ct1}$ also differs among the samples; however, it mainly reflects the surface-film response and high-frequency interfacial processes, and its contribution to the overall corrosion reaction is less significant than that of the low-frequency branch [33]. Therefore, the evaluation of corrosion resistance should primarily be based on the low-frequency impedance behavior and $R_{ct2}$, rather than solely on the magnitude of $R_{ct1}$.

In addition, compared with the pure 316L cladding layer, the corrosion resistance of the TiC-reinforced coatings does not continuously improve with increasing laser power, but instead shows an initial increase followed by a decrease. This indicates that the effect of TiC particles on corrosion resistance is dual in nature. On the one hand, an appropriate amount of uniformly distributed TiC particles is beneficial for improving microstructural compactness, enhancing surface-layer stability, and thus increasing corrosion resistance. On the other hand, excessively high laser heat input may aggravate interfacial mismatch around TiC particles, elemental segregation, and local electrochemical heterogeneity, thereby leading to a deterioration in corrosion resistance. Therefore, for the 5.0 wt.% TiC/316L cladding layer, a laser power of 1200 W appears to be the optimal condition for improving the overall corrosion resistance of the coating.

Combined with the microstructural observations, it can be seen that the coatings are mainly composed of an austenitic matrix, a small amount of undissolved TiC particles, and reprecipitated nanoscale TiC-rich particles, with the TiC-rich particle regions accompanied by noticeable Mo enrichment. At a moderate laser power, the TiC particles undergo an appropriate degree of dissolution and reprecipitation, leading to the formation of fine, dispersed, and relatively uniformly distributed TiC-rich particles. Meanwhile, the associated Mo enrichment is also more uniformly distributed in these regions, which is conducive to the formation of a Cr/Mo-enriched passive film with improved compactness and repassivation capability, thereby enhancing the low-frequency impedance response and improving the resistance to Cl^−^ attack [40,41]. In contrast, at a lower laser power, insufficient dissolution of TiC results in a higher fraction of undissolved particles and coarse particle/matrix interfaces, which are prone to act as preferential sites for localized corrosion initiation. At a higher laser power, however, overheating of the molten pool intensifies the redistribution and interfacial segregation of Ti, C, and Mo during the final stage of solidification, giving rise to more pronounced compositional fluctuations and micro-electrochemical heterogeneity near the TiC-rich particle/austenite interfaces. This effect may also be accompanied by higher residual stress and more interfacial defects, thereby weakening passive film stability and deteriorating corrosion resistance [42]. Therefore, based on the EIS results, the corrosion resistance of the samples can be ranked as follows: P12 > 316L ≳ P10 >> P14 > P16. These results indicate that, within the investigated processing window, a moderate laser power enables optimized control over the dissolution-reprecipitation behavior of TiC particles and solute redistribution during solidification, thereby yielding the most uniform microstructure and the best corrosion resistance.

### 3.6. Potentiodynamic Polarization Analysis of the 316L/TiC Composite Coatings

**Figure 12:**
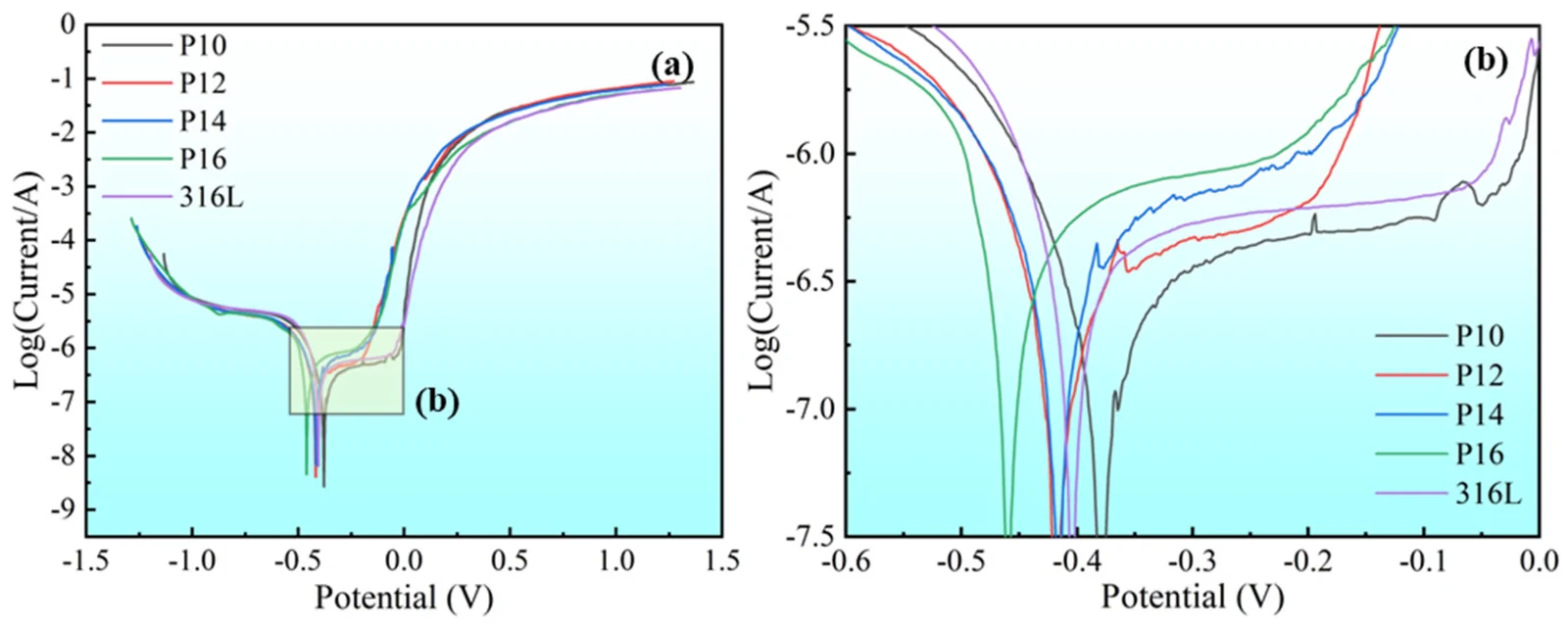
Potentiodynamic polarization curves of 316L/TiC composite coatings under different laser powers: (**a**) Full-range polarization curves; (**b**) Magnified partial region of polarization curves.

Potentiodynamic polarization is a commonly used method for evaluating the corrosion resistance of alloys, as it can directly reveal their electrochemical response characteristics. To further assess the corrosion behavior of the 5.0 wt.% TiC/316L cladding layers fabricated under different laser powers in 3.5 wt.% NaCl solution, potentiodynamic polarization tests were carried out on all samples. The corresponding results are shown in Figure 12, and the fitted electrochemical parameters are summarized in Table 3. As can be seen, all samples exhibit a distinct activation–passivation transition, indicating that each cladding layer is capable of forming a passive film to a certain extent in the chloride-containing medium. However, significant differences are observed among the samples in terms of corrosion current density, passivation stability, and pitting behavior, suggesting that laser power has a pronounced influence on the corrosion resistance of the coatings.

In terms of corrosion current density, $I_{corr}$, the values for P10, P12, P14, P16, and 316L are $2.51\times 10^{-7}$, $2.93\times 10^{-7}$, $4.22\times 10^{-7}$, $6.36\times 10^{-7}$ and $4.20\times 10^{-7}\;A/cm^{2}$, respectively. Among them, P10 exhibits the lowest $I_{corr}$, followed by P12, indicating that these two samples have lower corrosion rates and better corrosion resistance. P14 is close to 316L, whereas P16 shows the highest $I_{corr}$, implying that it is the most susceptible to corrosion and has the poorest corrosion resistance. In general, a lower $I_{corr}$ corresponds to a lower dissolution rate in the corrosive medium. Therefore, from the perspective of uniform corrosion, the corrosion resistance of the samples can be ranked as follows: P10 > P12 > 316L ≈ P14 > P16.

The corrosion potential, $E_{corr}$, indicates that there are certain differences in the spontaneous corrosion tendency among the samples, although its variation trend is not fully consistent with that of $I_{corr}$. Specifically, P10 and 316L exhibit relatively more noble $E_{corr}$ values, whereas P14 shows the most negative value, suggesting a relatively stronger thermodynamic tendency toward corrosion. However, $E_{corr}$ only reflects the tendency for corrosion initiation and cannot be used alone as the sole criterion for evaluating corrosion resistance. Therefore, it should be interpreted together with corrosion current density and passivation behavior.

The locally enlarged polarization curves in Figure 12b show that all samples display passivation behavior within a certain potential range, although the stability of the passive film differs markedly. After the activation–passivation transition, P10 and P12 exhibit relatively low and stable anodic current densities, indicating that the passive films formed on their surfaces are denser and more stable, and can effectively inhibit further attack of the corrosive medium on the substrate [43,44]. By contrast, P14 and especially P16 show higher current densities in the passive region and more pronounced fluctuations in the curves, indicating a weaker protective capability of the passive film and a greater tendency for local film breakdown and repassivation. The 316L sample also exhibits a certain degree of passivation behavior; however, considering its corrosion current density, its overall corrosion resistance remains inferior to that of P10 and P12.

The pitting potential, $E_{pit}$ and the passive interval, ΔE, can be used to evaluate the resistance of the samples to localized corrosion [39,45]. The results show that P10 has the highest pitting potential, −0.03V, which is significantly higher than those of P12, P14, and P16, and slightly higher than that of 316L. This indicates that the passive film on P10 is less susceptible to local breakdown in the chloride-containing medium and thus possesses superior resistance to pitting corrosion. The $E_{pit}$ of 316L is also relatively high, whereas P12 and P16 exhibit lower pitting potentials, suggesting that local destabilization of the passive film may occur at lower anodic potentials in these two samples. Meanwhile, 316L and P10 display relatively wide passive intervals, indicating that their passive films remain stable over a broader potential range. These results suggest that although P12 exhibits a relatively low corrosion current density and good overall corrosion resistance, its resistance to pitting corrosion is not the best. In contrast, P10 performs more favorably in suppressing both uniform corrosion and pitting corrosion.

Combined with the microstructural observations, the coatings are found to consist mainly of an austenitic matrix, a small fraction of undissolved TiC particles, and reprecipitated TiC-rich particles, with the TiC-rich particle regions accompanied by Mo enrichment. For the P10 sample, under the relatively low laser power, the TiC particles undergo only limited dissolution, resulting in relatively mild solute redistribution and weaker compositional gradients as well as reduced micro-electrochemical heterogeneity near the particle/matrix interfaces. Meanwhile, the presence of a small amount of dispersed TiC-rich particles together with moderate Mo enrichment is beneficial to the formation of a stable passive film, thereby leading to the lowest corrosion current density and the highest pitting potential [25]. In the P12 sample, the dissolution of TiC and the reprecipitation of TiC-rich particles are more pronounced, and the Mo enrichment contributes positively to the formation of a Cr/Mo-enriched passive film, so that the overall corrosion rate remains at a relatively low level [40]. However, with the increase in the number of TiC-rich particle/austenite heterogeneous interfaces, local compositional fluctuations and interfacial activation effects become more significant, making the passive film more susceptible to local breakdown during anodic polarization and thus resulting in a reduced pitting potential [42]. As the laser power is further increased to P14 and P16, overheating of the molten pool intensifies the redistribution and interfacial segregation of Ti, C, and Mo during the terminal stage of solidification, leading to increased microstructural heterogeneity, higher residual stress, and more interfacial defects, which in turn weaken the stability of the passive film and accelerate the corrosion process [42]. In particular, for P16, the beneficial effect of Mo enrichment is no longer sufficient to offset the adverse influence arising from heterogeneous interfacial activation and microstructural nonuniformity, and therefore this sample exhibits the poorest corrosion resistance.

It should be noted that there is a slight discrepancy between the corrosion-resistance rankings derived from EIS and potentiodynamic polarization, which is closely related to the different probing principles of the two methods and to the multiphase heterogeneous microstructure of the coatings. EIS applies a small-amplitude AC perturbation near the corrosion potential and mainly reflects passive-film stability, interfacial charge-transfer resistance, and mass-transport behavior of the corrosive medium. It is therefore more sensitive to the overall protective capability of the surface film and the interfacial uniformity of the coating. In contrast, potentiodynamic polarization scans over a wider potential range and actively accelerate anodic dissolution and film breakdown, making it more sensitive to the activation of local weak regions, pit initiation, and repassivation capability [46]. For the 316L/TiC composite coatings, the microstructure consists of an austenitic matrix, a small amount of undissolved TiC particles, and a large number of reprecipitated nanoscale TiC-rich particles. Uniformly dispersed nanoscale TiC-rich particles are beneficial for grain refinement, densification, and the formation of a stable passive film, and therefore contribute to higher impedance in EIS measurements. However, undissolved TiC particles and particle/matrix interfaces may also introduce electrochemical heterogeneity, making them more prone to local film breakdown and preferential dissolution under polarization conditions. Therefore, although the EIS and polarization results show slight differences in the detailed ranking, both consistently indicate that the corrosion resistance is jointly controlled by the “overall protective effect of the passive film” and the “local activation effect at heterogeneous interfaces.” The EIS results show that P12 possesses a higher interfacial charge-transfer resistance and a more favorable low-frequency impedance response, indicating stronger surface-film stability and better overall barrier performance. In contrast, the potentiodynamic polarization results indicate that P10 has a lower corrosion current density and a higher pitting potential, reflecting superior instantaneous corrosion suppression and better resistance to pitting corrosion. Overall, both P10 and P12 exhibit excellent corrosion resistance, although P10 is more advantageous in reducing corrosion rate and improving pitting resistance, whereas P12 is more favorable in enhancing interfacial film stability and the overall impedance response. As the laser power is further increased to 1400 W and 1600 W, the corrosion resistance of the coatings decreases markedly, with P16 showing the poorest performance.

## 4. Conclusions

In this study, 5.0 wt.% TiC-reinforced 316L composite coatings were fabricated by laser melting deposition under different laser powers, and the influence of laser power on microstructure evolution and overall performance was systematically evaluated. Based on the present results, the main conclusions can be drawn as follows:

(1) The coatings mainly consisted of an austenitic matrix, accompanied by a small amount of retained undissolved TiC particles and a large number of fine TiC-rich reprecipitated features. Laser power strongly affected the distribution and evolution of these heterogeneous reinforcing phases, thereby altering the degree of microstructural refinement and interfacial heterogeneity in the coatings.
(2) The introduction of TiC significantly improved the hardness and wear resistance of the 316L coating. The performance enhancement can be reasonably associated with the combined effects of residual TiC particle reinforcement, fine-particle dispersion strengthening, and matrix refinement. However, excessive laser power tended to increase microstructural nonuniformity and interfacial instability, which was unfavorable for further performance optimization.
(3) Electrochemical measurements showed that laser power had a pronounced influence on the corrosion behavior of the composite coatings. An appropriate laser power led to a denser and more uniform microstructure, lower corrosion current density, and improved overall corrosion resistance, whereas excessively high heat input increased local heterogeneity and degraded corrosion performance.
(4) The corrosion response of the coatings should be understood as the result of two competing effects: the overall protective role of the passive film and the local activation tendency associated with particle/matrix heterogeneity. From this perspective, the slight discrepancy between the corrosion rankings obtained from EIS and potentiodynamic polarization can be rationally interpreted.
(5) Overall, laser power is a key processing parameter for regulating the heterogeneous TiC-containing microstructure and balancing the mechanical and corrosion properties of LMD-fabricated 316L/TiC composite coatings. Among the investigated conditions, the coating fabricated at 1200 W showed the most favorable overall performance, particularly in terms of microstructural uniformity, impedance response, and the balance between wear and corrosion resistance.

It should be noted that the identification of the fine TiC-rich reprecipitated features in this work is based mainly on microstructural morphology, local elemental distribution, and consistency with related literature. More direct crystallographic verification and quantitative nanoscale interfacial characterization would require future high-resolution TEM-based analysis.

## Figures and Tables

**Table 1:** Chemical composition of 316L stainless steel powder (wt.%).

Element	C	Cr	Ni	Mo	Mn	Si	S	P	Fe
316L	0.01	16.76	10.56	2.50	0.58	0.37	0.005	0.018	Bal.

**Table 2:** Fitting results of EIS for all coating specimens tested in 3.5 wt.% NaCl solution.

Samples	$R_{s}\;(\Omega {cm}^{2})$	$R_{ct1}\;(\Omega {cm}^{2})$	$R_{ct2}\;(\Omega {cm}^{2})$	CPE _1_		CPE _2_		$\chi^{2}$
				n _1_	Y _1 (F cm^−2^)_	n _2_	Y _2 (F cm^−2^)_	
P10	3.56	10.31	5.33 × 10^5^	0.89	3.20 × 10^−7^	0.87	1.39 × 10^−5^	6.9 × 10^−4^
P12	3.05	9.98	6.12 × 10^5^	0.85	5.72 × 10^−7^	0.82	1.52 × 10^−5^	2.2 × 10^−3^
P14	3.66	10.49	1.61 × 10^5^	0.94	1.80 × 10^−7^	0.84	2.64 × 10^−5^	1.1 × 10^−3^
P16	3.85	14.31	0.56 × 10^5^	0.97	6.9 × 10^−8^	0.86	1.93 × 10^−5^	2.3 × 10^−3^
316L	1.91	23.06	5.55 × 10^5^	0.72	2.63 × 10^−6^	0.78	1.36 × 10^−5^	2.8 × 10^−3^

**Table 3:** Fitting results of potentiodynamic polarization curves for all coating specimens tested in 3.5 wt.% NaCl solution.

Sample	$I_{corr}$/(A/cm^2^)	$E_{corr}$/V	$E_{pass}$	$E_{pit}$/V	∆E
P10	2.51 × 10^−7^	−0.368	−0.314	−0.03	0.284
P12	2.93 × 10^−7^	−0.390	−0.357	−0.194	0.163
P14	4.22 × 10^−7^	−0.410	−0.368	−0.159	0.209
P16	6.36 × 10^−7^	−0.383	−0.377	−0.195	0.182
316L	4.20 × 10^−7^	−0.369	−0.344	−0.047	0.297

## Data Availability

The original contributions presented in this study are included in the article. Further inquiries can be directed to the corresponding authors.
